# A new integrated machine learning model: application to improve the accuracy of predicting left atrial appendage thrombus in patients with non-valvular atrial fibrillation

**DOI:** 10.3389/fmed.2025.1661696

**Published:** 2025-08-15

**Authors:** Peipei Mai, Huanhuan Huo, Xiaona Li, Dingwen Zhou, Fang He, Yongxin Li, Hua Wang

**Affiliations:** ^1^Department of Ultrasonography, Luoyang Central Hospital Affiliated to Zhengzhou University, Luoyang, China; ^2^Department of Ultrasound, The Second Affiliated Hospital, Medical School of Xi’an Jiaotong University, Xi’an, China; ^3^Division of Graduate, Xinxiang Medical University, Xinxiang, China; ^4^Department of Research and Development, Yizhun Medical AI Co. Ltd., Beijing, China

**Keywords:** non-valvular atrial fibrillation, transthoracic echocardiography, left atrial appendage thrombosis, machine learning models, transesophageal echocardiography

## Abstract

**Background:**

Non-Valvular Atrial fibrillation (NVAF) and atrial flutter are significant contributors to left atrial appendage thrombus (LAAT) formation. This study explores the potential of machine learning (ML) models integrating transthoracic echocardiography (TTE) and clinical data for non-invasive LAAT detection and risk assessment.

**Methods:**

A total of 698 patients with NVAF was recruited from Luoyang Central Hospital between January 2021 and May 2024, including 558 patients for retrospective analysis and 140 for prospective validation. Based on transesophageal echocardiography (TEE) results, patients were categorized into three groups: non-thrombotic, blood stasis, and thrombotic. Four ML algorithms—Random Forest, Logistic Regression (LR), Support Vector Machine, and XGBoost—were developed using TTE and clinical data to predict LAAT.

**Results:**

Univariate analysis identified significant predictors of LAAT, including permanent AF, heart failure, BNP, uric acid, D-dimer, mitral regurgitation, LVEF, LVED, LAD, CHA₂DS₂-VASc score, and LAA velocity (*p* < 0.05). The combined TTE data model outperformed independent TTE-based models but was slightly less accurate than the TEE model. Among ML algorithms, the LR model demonstrated the best performance, achieving an area under the curve (AUC) of 80.9% in the test set and 78.7% in prospective validation for the thrombotic state group. For the thrombotic group, the LR model achieved an AUC of 80.0%, closely approaching the TEE model’s 84.0%.

**Conclusion:**

The LR model provides a reliable non-invasive approach for LAAT screening in high-risk AF patients by integrating TTE features with clinical data, potentially reducing reliance on TEE.

## 1 Introduction

Non-valvular Atrial fibrillation (NVAF) and atrial flutter are significant contributors to the formation of left atrial appendage thrombus (LAAT), leading to an increased risk of thromboembolic events. It has been established that LAAT is often identified as a source of thrombus formation in patients who have recently experienced strokes, highlighting the critical need for effective detection methods (1–3). Despite the complexity of stroke mechanisms, LAAT is frequently implicated in thromboembolic complications, particularly in patients undergoing cardioversion (4, 5).

The restoration of sinus rhythm is associated with a heightened risk of thromboembolic events, as embolization of pre-existing thrombi in the atrium is a plausible cause of such incidents (6). Consequently, current guidelines contraindicate the performance of cardioversion and catheter ablation in the presence of LAAT (7, 8). Although oral anticoagulation (OAC) reduces the risk of LAAT formation, it does not eliminate this risk entirely, necessitating alternative detection methods (9–11). Transesophageal echocardiography (TEE) is recognized as the gold standard for LAT detection, demonstrating high sensitivity and specificity (12, 13). However, its associated discomfort, complexity, and costs pose significant challenges for routine clinical application, leading to an unmet clinical need for a more accessible and practical risk assessment method for LAAT (14–16).

Transthoracic echocardiography (TTE) presents a less invasive and more cost-effective alternative to TEE, allowing for rapid assessment. Several studies have identified TTE features that correlate with the presence of LAAT; however, there has been a lack of research integrating clinical and TTE data to create a personalized risk assessment model (17, 18). In response to these limitations, A novel prediction model for LAAT detection has been developed in this study, integrating TTE measurements with clinical data. Machine learning algorithms, including Support Vector Machine (SVM), Random Forest (RF), XGBoost (XGB), and Logistic Regression (LR), were harnessed to construct and validate the model. The model’s criteria for thrombus prediction were based on left atrial appendage (LAA) emptying velocity and LAA filling velocity, with TEE as the reference standard. This approach aims to improve the accuracy and practicality of LAAT risk assessment by comparing the performance of the machine learning-based model to traditional methods.

This study aims to establish a reliable and non-invasive predictive tool for LAAT risk by leveraging machine learning algorithms on readily available clinical and TTE data, thereby potentially reducing the reliance on invasive TEE procedures and enhancing patient management strategies.

## 2 Methods

### 2.1 Study population

This study included patients from Luoyang Central Hospital between January 2021 and December 2023. The inclusion criteria were: 1. patients with an initial diagnosis of AF upon discharge, 2. patients who underwent TEE for assessment of left atrial appendage (LAA) parameters, and TTE for cardiac measurements, and 3. atrial fibrillation duration ≥48 h. The exclusion criteria were: 1. patients with moderate or severe mitral stenosis with AF, and 2. patients with AF after mechanical valve replacement (e.g., aortic or mitral valve replacement).

**Figure 1:**
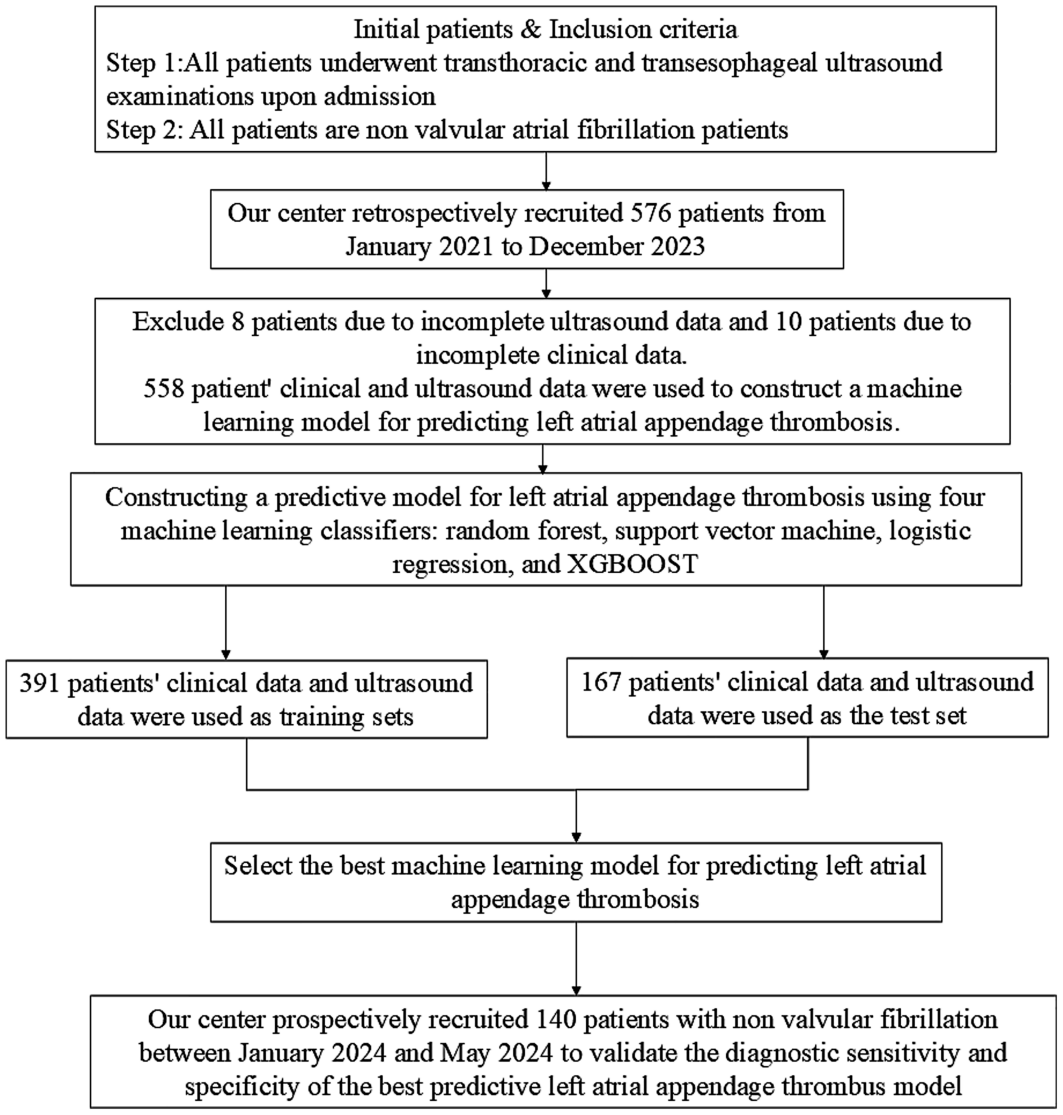
Flowchart of inclusion and exclusion criteria for non valvular atrial fibrillation patients in this study.

Both retrospective and prospective analyses were conducted based on the most recent hospitalization records. Data on LAAT and cardiac measurements from TTE were collected. Any patient data that did not meet the inclusion criteria were excluded from the analysis. A total of 698 patients were enrolled in the study, of which 558 were included in the retrospective analysis, and 140 were used for prospective validation of the best-performing machine learning models (Figure 1). Patients were divided into three groups based on TEE results: no thrombus group (first), thrombus state group (second), and thrombus group (third).

Ethical approval for the study (Approval No. LWLL-2024-11-05-02) was obtained from the Ethics Committee of Luoyang Central Hospital, affiliated with Zhengzhou University, prior to the commencement of the study. Written informed consent was obtained from all patients.

### 2.2 Data collection

Clinical baseline data were collected from patients, including but not limited to age, sex, weight, and medical history. All echocardiographic examinations were conducted by experienced ultrasound physicians in accordance with the guidelines established by the American Society of Echocardiography (ASE). Imaging data from both TTE and TEE were recorded, including parameters such as left atrial size, left ventricular size, left ventricular ejection fraction (LVEF), degree of mitral regurgitation, LAA emptying velocity, and LAA filling velocity.

### 2.3 Machine learning model development and prospective validation

Due to the extensive feature set and limited sample size, a feature selection technique was utilized to identify an optimal subset of features from the initial set. The process began with applying min-max normalization to all features in the training data. Subsequently, to remove irrelevant and redundant features, a two-sample *t*-test was conducted to identify features exhibiting significant differences (*p* < 0.05) between the MSA-P and IPD groups. For nodal graph metrics, False Discovery Rate (FDR) correction was implemented, while for FC matrices, Network-Based Statistic (NBS) correction was applied. Following this, a random forest (RF) classifier with 10-fold cross-validation was employed to identify a subset of the most informative features from the high-dimensional feature space. To prevent data leakage during the feature selection process, each fold of the 10-fold cross-validation was independently partitioned. Min-max normalization and feature selection using Random Forest were strictly applied within each training subset. The RF algorithm assigned a rank to each feature based on its importance in each iteration, and the top 10 features with the highest importance scores were chosen. These selections were recorded as indices or names of the chosen features. A tally was kept of how often each feature was selected, and the top 10 features that were selected most frequently were retained. Lastly, Spearman’s rank correlation coefficient was employed to assess the relationships among the remaining connectome features. If the absolute value of the correlation coefficient was at least 0.7 and the *p* value was less than 0.05, the feature with the lower importance was eliminated.

**Figure 2:**
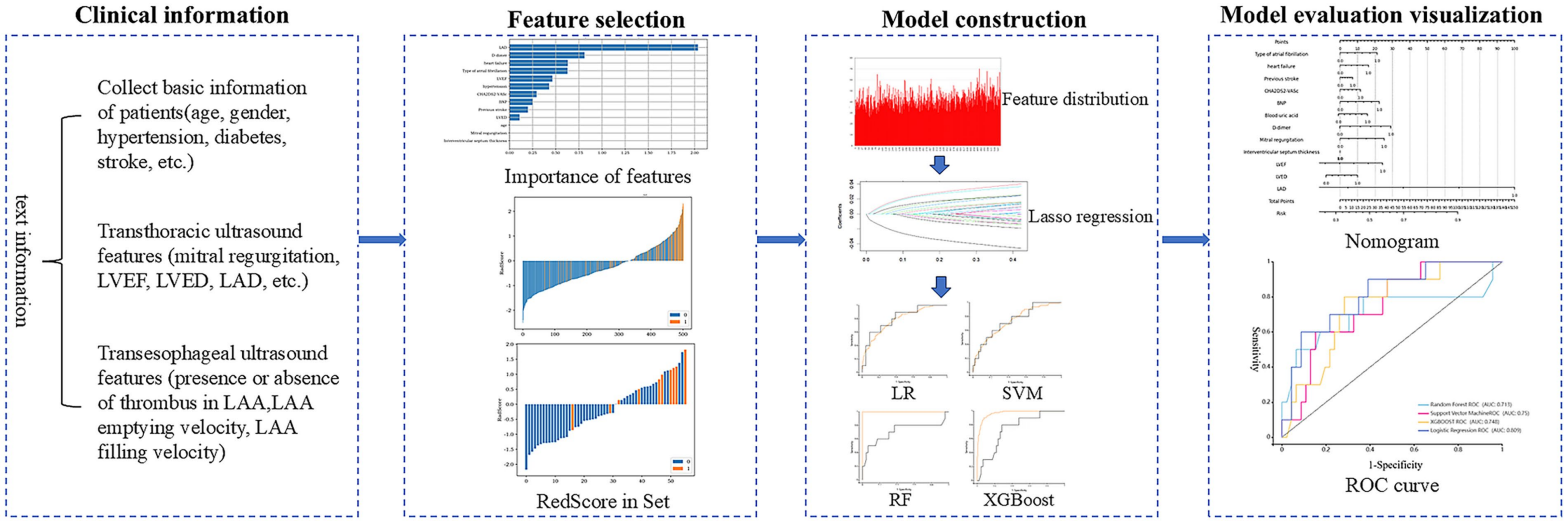
Process diagram for predicting left atrial appendage thrombus using machine learning combined with clinical data and transthoracic ultrasound features.

Following the feature selection process, we employed supervised machine learning techniques to develop a classification model. For this research, the dataset included in this study has very few missing data (less than 5%), and estimates were made using the median of continuous variables and the mode of categorical variables. We conducted model training using Random Forest (RF), Logistic Regression (LR), Support Vector Machine (SVM), and eXtreme Gradient Boosting (XGBoost) on the subjects within the training dataset. The optimal hyperparameters for these models were determined through a grid search approach. The retrospective dataset was partitioned into a training set (80%) and test set (20%) using stratified random sampling. Hyperparameter tuning was conducted through a nested cross-validation process exclusively on the training set, employing a grid search strategy to prevent overfitting. The models were trained on critical features encompassing demographic information, clinical records, NVAF subtypes, structural and functional measurements of the left atrial diameter (LAD) and left atrial appendage (LAA), laboratory data, and treatment variables. We then plotted the Receiver Operating Characteristic (ROC) curves for these models. Performance metrics such as the Area Under the Curve (AUC), precision, recall, specificity, positive predictive value (PPV), and negative predictive value (NPV) were computed to assess the efficacy of each model on both the training and validation datasets Figure 2.

For internal prospective validation, the final model was independently evaluated using a prospective validation cohort (*n* = 140).we employed 140 patients collected from our center between January 2024 and May 2024. This cohort was used to assess the clinical relevance and applicability of the optimal machine learning models, confirming their effectiveness in a clinical setting.

### 2.4 Statistical analysis

Statistical analyses were conducted utilizing IBM SPSS Statistics for Windows, Version 25.0 (IBM Corp.). The Shapiro–Wilk test assessed the normality of the data distribution. When data followed a normal distribution, parametric tests were applied; otherwise, non-parametric tests were employed. Categorical variable differences were evaluated using the chi-squared test, while continuous variable differences were assessed with either the independent *t*-test or the Mann–Whitney *U* test, depending on the data distribution. A univariate analysis was performed to determine factors correlated with left atrial appendage thrombosis (LAT).

To evaluate the diagnostic performance of transthoracic echocardiography (TTE) features, receiver operating characteristic (ROC) curves were generated, and the area under the curve (AUC) was calculated. Multivariable logistic regression analysis was performed to identify independent predictors of LAAT, adjusting for potential confounders identified in univariate analysis. Statistical significance was set at a two-tailed *p*-value of <0.05.

## 3 Results

### 3.1 Univariate analysis of clinical data

In the comparative analysis of clinical data between first Group (non-thrombus) and Group second and third (thrombus status group) Table 1, univariate analysis identified significant associations with the following variables: permanent atrial fibrillation (AF), heart failure, B-type natriuretic peptide (BNP), uric acid, and D-dimer, Mitral regurgitation, LVEF, LVED, LAD, CHA2DS2-VASc score, Left atrial appendage filling velocity, Left atrial appendage emptying rate with all showing statistical significance (*p* < 0.05). Similarly, in the comparison between Group first and Group second (thrombus group) Table 1, the variables of permanent AF, heart failure, history of stroke, BNP, uric acid, and D-dimer, Mitral regurgitation, LVEF, LVED, LAD, CHA2DS2-VASc score, Left atrial appendage filling velocity, Left atrial appendage emptying rate also demonstrated statistical significance (*p* < 0.05). These findings are consistent with previous research on the risk factors associated with LAAT.

**Table 1:** Groups comparison of clinical and ultrasound features.

Characteristics	Groups			Thrombus state vs. Non-thrombotic			Thrombus vs. Non-thrombotic		
	Thrombus state (*n* = 102)	Thrombus (*n* = 56)	Non-thrombotic (*n* = 456)	x^2^/t	*p*	OR [95% CI]	x^2^/t	*p*	OR [95% CI]
Gender, Female (%)	47 (46.07)	23 (41.07)	206 (45.17)	0.00	0.956	1.04 (0.69, 1.56)	0.340	0.560	0.85 (0.49, 1.47)
Radiofrequency ablation, Yes (%)	9 (8.82)	3 (5.30)	26 (5.7)	0.84	0.342	1.60 (0.77, 3.32)	0.011	0.916	0.94 (0.34, 2.38)
Electrical cardioversion, Yes (%)	2 (1.96)	1 (1.78)	12 (2.63)	0.00	0.967	0.74 (0.18, 2.98)	0.144	0.704	0.67 (0.09, 5.01)
Medication, Yes (%)	24 (23.52)	13 (23.21)	128 (28.07)	0.67	0.414	0.79 (0.48, 1.31)	0.589	0.443	0.78 (0.43, 1.43)
Types of atrial fibrillation, paroxysmal (%)	49 (48.03)	28 (50.00)	132 (28.94)	12.43	<0.001	2.34 (1.44, 3.80)	10.289	0.001	2.45 (1.51, 3.98)
Hypertension, Yes (%)	57 (55.88)	36 (64.28)	233 (51.09)	0.55	0.456	1.21 (0.80, 1.83)	3.42	0.064	1.72 (1.05, 2.83)
Heart failure, Yes (%)	43 (42.15)	24 (42.85)	91 (19.95)	19.33	<0.001	2.92 (1.83, 4.66)	14.937	<0.001	3.00 (1.76, 5.12)
Diabetes, Yes (%)	16 (15.68)	10 (17.85)	69 (15.13)	0.00	0.991	1.04 (0.58, 1.86)	0.284	0.594	1.22 (0.76, 1.97)
Coronary heart disease, Yes (%)	48 (47.05)	30 (53.57)	201 (44.07)	0.19	0.662	1.13 (0.77, 1.65)	1.815	0.178	1.46 (0.89, 2.40)
Stroke, Yes (%)	25 (24.50)	16 (28.57)	78 (17.10)	2.43	0.119	1.57 (0.94, 2.64)	4.375	0.036	1.89 (1.13, 3.17)
Smoking, Yes (%)	16 (15.68)	11 (19.64)	103 (22.58)	2.08	0.149	0.64 (0.37, 1.08)	0.250	0.617	0.84 (0.49, 1.43)
Drinking, Yes (%)	9 (8.82)	4 (7.14)	22 (4.82)	1.65	0.198	1.91 (0.88, 4.16)	0.556	0.456	1.52 (0.51, 4.53)
Age, Year	66 (58, 71)	68.00 (58.50, 72.00)	64 (56.25, 71)	1.423	0.155	1.66 (1.06, 2.59)	1.551	0.121	2.04 (1.23, 3.38)
Atrial fibrillation duration, (months)	12 (1.00, 51.00)	12.00 (1.25, 36.00)	12 (1.00, 48.00)	0.781	0.435	2.00 (1.00, 3.99)	0.492	0.623	1.58 (0.94, 2.66)
BMI (Kg/m^2^)	25.62 (24.31, 26.67)	25.54 (23.77, 26.30)	25.62 (24.15, 26.67)	0.400	0.689	1.82 (1.20, 2.76)	0.351	0.726	0.56 (0.32, 1.00)
BNP (pg/ml)	515.56 (107.06, 1072.00)	603.68 (105.19, 1198.00)	153.59 (54.25, 716.44)	4.282	<0.001	2.56 (1.61, 3.88)	3.336	<0.001	2.84 (1.68, 4.80)
Blood uric acid (umol/L)	348.00 (295.50, 432.50)	343.00 (278.50, 448.25)	332.00 (267.50, 390.00)	2.772	0.006	2.33 (1.49, 3.65)	1.783	0.075	2.58 (1.56, 4.25)
D-Dimer (mg/L)	300.00 (160.00, 695.00)	300.00 (142.50, 725.00)	190.00 (140.00, 330.00)	3.485	<0.001	2.79 (1.78, 4.36)	2.541	0.011	3.67 (2.14, 6.28)
Prothrombin time (inr)	12.30 (11.20, 15.05)	12.25 (11.20, 15.28)	12.20 (11.10, 14.20)	1.130	0.259	2.20 (1.44, 3.63)	1.131	0.258	2.77 (1.46, 5.27)
Mitral regurgitation (cm^2^)	3.40 (1.80, 6.20)	3.35 (1.35, 6.38)	2.00 (1.00, 4.50)	3.344	<0.001	2.68 (1.67, 4.25)	2.304	0.021	2.26 (1.34, 3.80)
Interventricular septum thickness (mm)	11.00 (10.00, 12.00)	11.10 (10.00, 12.00)	10.00 (9.00, 11.00)	2.459	0.014	1.78 (1.05, 2.97)	2.903	0.004	2.59 (1.55, 4.31)
LVEF (%)	60.00 (50.75, 65.00)	62.00 (51.25, 65.75)	63.00 (59.00.67.00)	3.559	<0.001	2.32 (1.46, 3.66)	2.246	0.025	2.21 (1.32, 3.69)
LVED (mm)	48.00 (45.00, 52.00)	48.00 (44.25, 52.00)	47.00 (43.00, 50.00)	2.991	0.003	2.56 (1.65, 3.95)	2.314	0.021	2.69 (1.51, 4.63)
LAD (mm)	46.00 (42.00, 53.00)	45.00 (41.00, 52.75)	41.00 (37.00, 45.00)	7.655	<0.001	5.41 (3.56, 8.05)	4.768	<0.001	3.82 (2.28, 6.39)
CHA2DS2-VASc	3.00 (1.75, 4.25)	3.00 (2.00, 5.00)	2.00 (1.00, 4.00)	2.514	0.012	1.69 (1.09, 2.66)	3.086	0.002	2.36 (1.39, 3.99)
Left atrial appendage filling velocity (cm/s)	30.00 (20.00, 42.00)	42.40 (22.40, 48.23)	47.6 (36.80, 61.70)	6.619	<0.001	5.93 (3.69, 9.54)	4.393	<0.001	9.33 (4.02, 21.7)
Left atrial appendage emptying rate (cm/s)	20.00 (14.00, 28.10)	30.00 (17.05, 41.64)	40.00 (27.80, 55.00)	8.107	<0.001	13.10 (6.41, 26.75)	4.697	<0.001	12.35 (4.93, 30.88)

BMI, Body Mass Index; BNP, Brain Natriuretic Peptide; LVEF, Left Ventricular Ejection Fraction; LVED, Left ventricular end diastolic; LAD, Left atrial diameter.

### 3.2 Diagnostic value of transthoracic echocardiography

To evaluate the diagnostic value of TTE features in predicting LAAT, we compared the diagnostic performance of a model utilizing combined TTE data with a model based on TEE data. In the non-thrombus vs. thrombus status group, the combined TTE diagnostic model demonstrated significantly higher sensitivity (65.7%), specificity (73.0%), and accuracy (71.7%) compared to the model using TTE alone. However, when compared to the combined TEE model, which exhibited sensitivity (80.3%), specificity (72.2%), and accuracy (73.4%), the comprehensive TTE model showed lower sensitivity, Table 2.

**Table 2:** Diagnostic performance of the model in predicting the risk of thrombotic state.

Models	SEN (%)	SPE (%)	ACC (%)	NPV (%)	PPV (%)	AUC (%) [95% CI]	Cut of value	*p* value *χ*^2^ test
MR (cm^2^)	70.6	51.1	54.7	88.6	24.4	0.61 (0.55, 0.67)	2.1	0.052
EF (%)	42.2	72.6	67.0	84.9	25.6	0.61 (0.55, 0.68)	58.5	0.005
LVED (mm)	36.2	81.7	72.2	85.1	30.8	0.59 (0.53, 0.66)	50.5	0.003
LAD (mm)	66.6	73.0	71.8	90.7	35.6	0.74 (0.69, 0.80)	44.5	<0.001
MR + EF + LVED+LAD	65.7	73.0	71.7	90.5	35.3	0.74 (0.68, 0.79)	0.2	<0.01
LAA filling velocity (cm/s)	57.7	81.3	77.7	91.5	35.7	0.75 (0.68, 0.81)	31.6	<0.001
LAA emptying velocity (cm/s)	86.6	67.4	70.3	96.5	32.4	0.81 (0.76, 0.87)	30.2	<0.001
LAA filling + emptying velocity (cm/s)	80.3	72.2	73.4	95.3	34.1	0.80 (0.75, 0.86)	0.2	<0.001

AUC area under the receiver operating characteristic curve, SEN sensitivity, SEP specifcity, ACC accuracy, NPV negative predictive value, PPV positive predictive value.

In the non-thrombus and thrombus groups, the combined TTE diagnostic model again revealed significantly higher sensitivity (50.0%), specificity (84.2%), and accuracy (80.5%) compared to the model using TTE alone. Conversely, the sensitivity (82.9%), specificity (72.4%), and accuracy (73.3%) of the combined TEE model were notably higher, indicating that the comprehensive TTE model has lower sensitivity in this context, Table 3.

**Table 3:** Diagnostic performance of the model in predicting the risk of thrombotic.

Models	SEN (%)	SPE (%)	ACC (%)	NPV (%)	PPV (%)	AUC (%) [95% CI]	Cut of value	*p* value *χ*^2^ test
MR (cm^2^)	71.4	51.2	53.4	93.6	15.3	0.60 (0.51, 0.68)	2.1	<0.001
EF (%)	41.1	76.0	72.2	91.3	17.4	0.59 (0.51, 0.68)	58.5	<0.001
LVED (mm)	37.5	81.7	76.9	91.4	20.1	0.59 (0.51, 0.68)	50.5	<0.001
LAD (mm)	58.9	73.0	71.4	93.5	21.1	0.70 (0.62, 0.78)	44.5	<0.001
MR + EF + LVED+LAD	50.0	84.2	80.5	93.2	28.0	0.74 (0.64, 0.83)	0.1	<0.001
LAA filling velocity (cm/s)	60.0	85.3	83.3	36.8	26.6	0.78 (0.70, 0.86)	48.3	<0.001
LAA emptying velocity (cm/s)	85.3	67.4	68.9	98.0	19.3	0.84 (0.77, 0.91)	41.7	<0.001
LAA filling + emptying velocity (cm/s)	82.9	72.4	73.3	97.9	21.0	0.84 (0.77, 0.91)	0.1	<0.001

AUC area under the receiver operating characteristic curve, SEN sensitivity, SEP specifcity, ACC accuracy, NPV negative predictive value, PPV positive predictive value.

### 3.3 Performance of machine learning models

**Figure 3:**
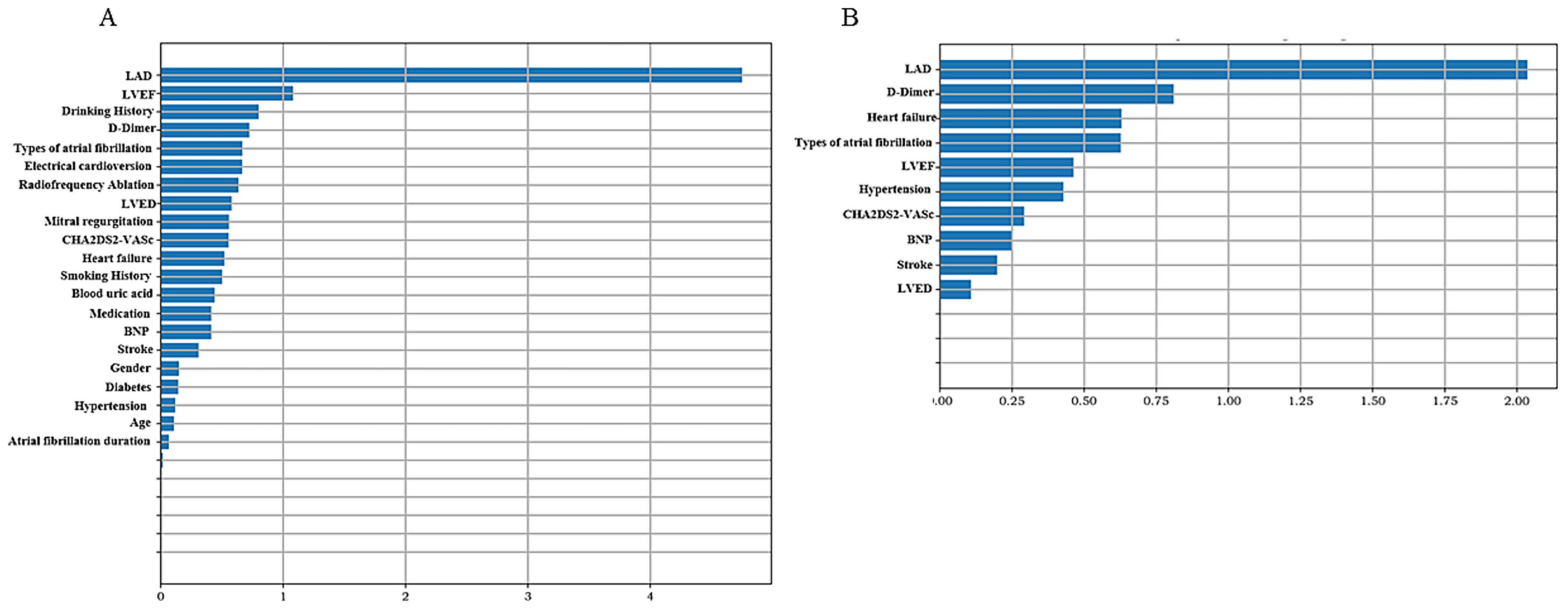
Summary chart of feature importance representative variables. The higher the variable value, the greater the contribution to predicting left atrial appendage thrombus status. **(A)** Non-thrombus vs. thrombus status group. **(B)** Non-thrombus vs. thrombus group.

Machine learning methods, including Random Forest (RF), Support Vector Machine (SVM), Logistic Regression (LR), and eXtreme Gradient Boosting (XGBoost), were utilized to develop predictive models for LAAT by integrating echocardiographic and clinical data. In the non-thrombus vs. thrombus status group, the LR model demonstrated superior sensitivity and accuracy across both training and testing datasets (*p* < 0.05). In the training set, the LR model achieved a sensitivity of 81.5%, specificity of 61.7%, accuracy of 65.3%, and area under the curve (AUC) of 78.5%. In the testing set, the model displayed a sensitivity of 60.0%, specificity of 89.1%, accuracy of 83.9%, and AUC of 80.9% (Table 4). Prospective validation using data from our center confirmed the stability and effectiveness of the LR model, yielding sensitivity of 82.4%, specificity of 61.6%, accuracy of 65.4%, and AUC of 78.7% (Table 4). The 10 most important features identified by the model included LAD, LVEF, Drinking History, D-dimer levels, type of atrial fibrillation, history of electrical cardioversion, radiofrequency ablation, left ventricular end-diastolic diameter (LVED), and degree of mitral regurgitation, Figure 3A.

**Table 4:** Model performance based on different machine learning algorithms for thrombus state group vs. non-thrombotic group: training set and test set; prospective validation performance of logistic regression models.

Model	558 retrospective modeling cases (first group = 456, second group = 102)								140 prospective validated cases (first group = 107, second group = 33)			
	Training set				Test set				Validation set			
	AUC (%)	SEN (%)	SPE (%)	ACC (%)	AUC (%)	SEN (%)	SPE (%)	ACC (%)	AUC (%) [95% CI]	SEN (%) [95% CI]	SPE (%) [95% CI]	ACC (%)
Logistic regression	78.5	81.5	61.7	65.3	80.9	60.0	89.1	83.9	78.7 (0.74, 0.83)	82.4 (0.74, 0.89)	61.6 (0.57, 0.66)	65.4
Support vector machine	74.5	78.3	57.3	61.2	75.0	60.0	82.6	78.6				
Random forest	100.0	100.0	100.0	100.0	71.3	50.0	93.5	85.7				
XGBOOST	97.0	90.2	92.4	94.0	74.8	80.0	71.7	73.2				

First group: non-thrombotic group, second group: thrombus state group.

In the non-thrombus vs. thrombus group, the LR model again exhibited the highest sensitivity and accuracy among the four machine learning methods (*p* < 0.05). The training set results showed a sensitivity of 68.0%, specificity of 70.2%, accuracy of 70.0%, and AUC of 73.2%. In the testing set, the model achieved sensitivity of 83.3%, specificity of 78.3%, accuracy of 78.8%, and AUC of 80.1% (Table 5). Prospective validation with our center’s data demonstrated robust performance, yielding sensitivity of 85.7%, specificity of 76.9%, accuracy of 77.9%, and AUC of 80.0% (Table 5). The 10 most important features for this model included LAD, D-dimer levels, heart failure status, type of atrial fibrillation, LVEF, history of hypertension, CHA2DS2-VASc score, BNP levels, history of previous stroke, and LVED, Figure 3B.

**Table 5:** Model performance based on different machine learning algorithms for thrombus state group vs. non-thrombotic group: training set and test set; prospective validation performance of logistic regression models.

Model	512 retrospective modeling cases (first group = 456, third group = 56)								121 prospective validated cases (first group = 107, third group = 14)			
	Training set				Test set				Validation set			
	AUC (%)	SEN (%)	SPE (%)	ACC (%)	AUC (%)	SEN (%)	SPE (%)	ACC (%)	AUC (%)	SEN (%) [95% CI]	SPE (%) [95% CI]	ACC (%)
Logistic regression	73.2	68.0	70.2	70.0	80.1	83.3	78.3	78.8	80.0 (0.68, 0.93)	85.7 (0.60, 0.96)	77.8 (0.69, 0.85)	78.7
Support vector machine	91.0	82.0	95.1	93.7	63.4	83.3	58.7	61.5				
Random Forest	99.6	100	93.4	94.1	71.9	66.7	84.8	82.7				
XGBOOST	98.6	94.0	94.6	94.6	65.6	50.0	95.7	90.7				

First group: non-thrombotic group, third group: thrombus group.

**Figure 4:**
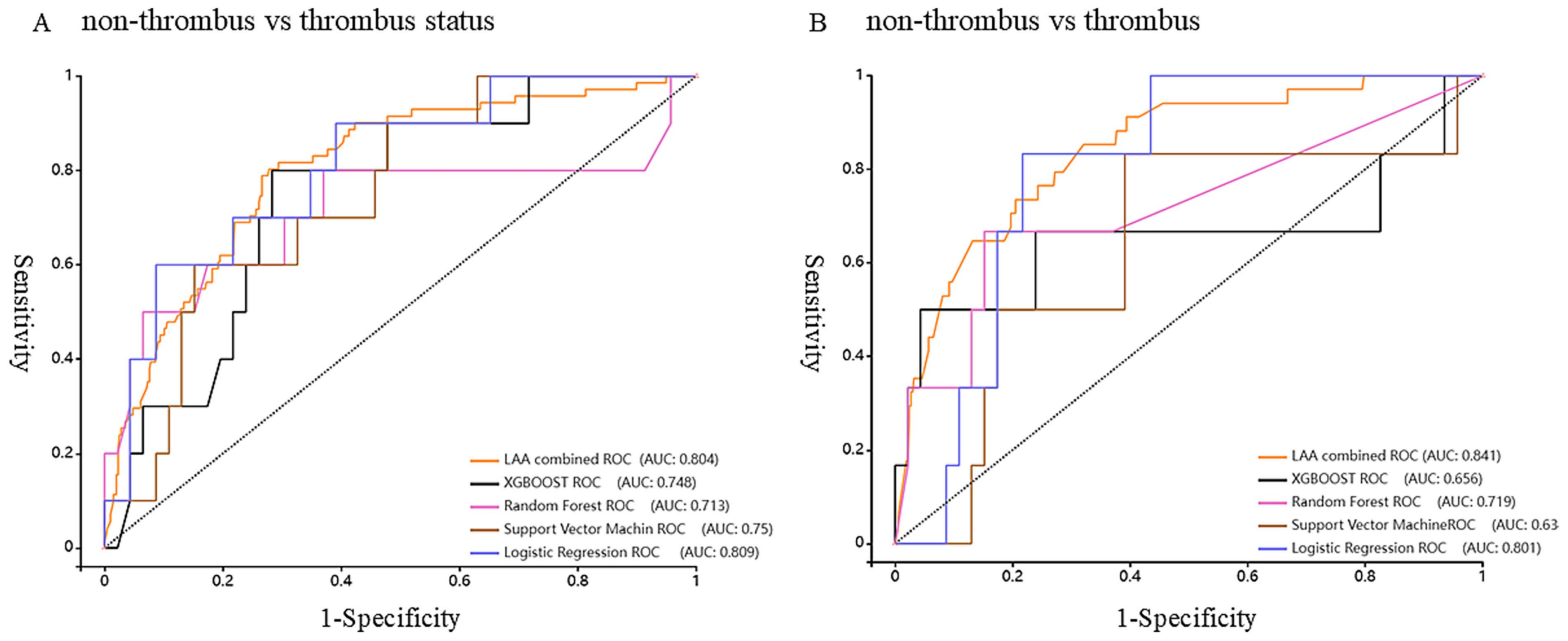
Receiver Operating Characteristic curve (ROC) of the model predicts the status and risk of left atrial appendage thrombosis. **(A)** Non-thrombus vs. thrombus status group. **(B)** Non-thrombus vs. thrombus group.

In the non-thrombus vs. thrombus status group, the LR model demonstrated a diagnostic sensitivity of 82.4%, specificity of 61.6%, accuracy of 65.4%, and an area under the curve (AUC) of 78.7%. When compared to the TEE combined prediction model, which had a sensitivity of 80.3%, specificity of 72.2%, accuracy of 73.4%, and AUC of 80.4%, the difference in AUC was not statistically significant. However, the LR model exhibited a higher sensitivity than the TEE model. This suggests that the LR model could be a useful alternative for detecting LAAT, particularly in scenarios where maximizing sensitivity is critical, such as in patients at high risk of thromboembolism, Figure 4A.

In the non-thrombus vs. thrombus group, the LR model showed an even higher diagnostic sensitivity of 85.7%, specificity of 76.9%, accuracy of 77.9%, and AUC of 80.0%. These values were compared with those from the TEE model, which had a sensitivity of 82.9%, specificity of 72.4%, accuracy of 73.3%, and AUC of 84.0%. The difference in AUC was statistically significant, indicating that the TEE model had better overall discriminatory power. However, the LR model’s higher sensitivity, specificity, and accuracy in this group highlights its potential as a robust non-invasive tool for diagnosing LAAT. The LR model’s ability to outperform TEE in terms of sensitivity, while maintaining competitive accuracy and AUC, reinforces its utility as a complementary diagnostic approach in clinical practice, Figure 4B.

## 4 Discussion

Non-valvular atrial fibrillation (NVAF) is one of the most common arrhythmias and is closely associated with the formation of LAA thrombosis. The incidence of LAA thrombosis is high in patients with atrial fibrillation, and it is often the primary source of stroke and other serious cardiovascular complications (19). The presence of LAA thrombosis is a critical factor in treatment decisions for AF patients, as its formation significantly increases the risk of embolic events, particularly in patients who are not on effective anticoagulation therapy (20). Therefore, early prediction and detection of LAA thrombosis are vital in preventing AF-related complications. While transesophageal TEE is the current gold standard for diagnosing LAA thrombosis, its invasiveness and technical requirements limit its widespread use in routine clinical screening. In contrast, TTE, a non-invasive and easy-to-use tool, holds great potential for routine screening in AF patients. However, the diagnostic accuracy of TTE alone is insufficient, and improving its predictive performance remains a challenge in clinical research. To address this issue, our study proposes an innovative approach that integrates machine learning with clinical data and TTE to non-invasively predict the risk of LAA thrombosis, aiming to provide a more convenient, accurate, and feasible screening tool for clinical practice.

The results of the univariate analysis revealed that several clinical and echocardiographic parameters showed significant statistical differences between the LAA thrombosis group, the thrombus status group, and the no-thrombus group, when compared to those without LAA thrombosis. These factors include atrial fibrillation type, heart failure, BNP, serum creatinine, D-dimer, mitral regurgitation, LVEF, LVED, LAD, CHA2DS2-VASc score, LAA emptying velocity, and LAA filling velocity. These findings are consistent with previous studies, further confirming the importance of these clinical and echocardiographic features in the formation of LAA thrombosis (21–23). Particularly, BNP, D-dimer, and LAA functional parameters such as emptying and filling velocities are closely related to thrombosis formation and hemodynamic status (24). Additionally, the CHA2DS2-VASc score, a traditional risk assessment tool for stroke in AF patients (25), also demonstrated strong predictive ability in this study. The statistical significance of these variables suggests that LAA thrombosis is influenced not only by cardiac structural and functional abnormalities but also by various hemodynamic and coagulation factors, thus providing a solid theoretical foundation for the development of predictive models.

In this study, both single and combined models using TTE and TEE demonstrated their potential in predicting LAA thrombosis and thrombus status. When compared to the no-thrombus group, the combined TTE model (including mitral regurgitation, LVEF, LVED, and LAD) showed improvements in diagnostic sensitivity, specificity, and AUC in the thrombus status group (sensitivity 65.7%, specificity 73.0%, AUC 0.74). The combined TEE model, based on LAA emptying and filling velocities, showed even higher diagnostic performance in the thrombus status group (sensitivity 80.3%, specificity 72.2%, AUC 0.80). Moreover, compared to the thrombus group, the diagnostic sensitivity, specificity, and AUC of the combined TEE model also showed significant improvements (sensitivity 82.9%, specificity 72.4%, AUC 0.84). These results not only align with existing research but also reaffirm the gold standard status of TEE for diagnosing LAA thrombosis. However, it is important to note that while TEE has stronger diagnostic capability, its invasiveness and technical demands make TTE a more practical and convenient screening tool, especially for routine clinical use. Therefore, by comparing these two methods, our study highlights the complementary nature of different echocardiographic techniques in thrombosis prediction, laying the groundwork for more refined modeling approaches to enhance diagnostic efficacy.

The study further incorporated four machine learning models—Logistic Regression, Support Vector Machine, Random Forest, and XGBoost—integrating clinical data and TTE features to develop a non-invasive predictive model for LAA thrombosis. Among these, the Logistic Regression model demonstrated superior performance, particularly in sensitivity and stability, compared to the other models. The LR model’s superior performance is likely due to its interpretability, lower complexity, and reduced susceptibility to overfitting compared to RF and XGBoost, particularly given our moderate sample size. Its higher sensitivity compared to TEE may result from integrating clinical data, compensating for echocardiographic limitations and enhancing diagnostic accuracy. In the thrombus group, the validation set achieved an AUC of 80.0%, with sensitivity at 85.7%, specificity at 76.9%, while in the thrombus status group, the AUC was 78.7%, with sensitivity of 82.4% and specificity of 61.6%. More importantly, in prospective validation, the sensitivity for the thrombus group increased by 3% compared to the combined TEE model, while the sensitivity for the thrombus status group increased by 4%. These results confirm the clinical feasibility of the Logistic Regression model, particularly its potential for enhancing early screening sensitivity and reducing the risk of embolic events, demonstrating its advantage in high-risk patient screening. The results not only validate the effectiveness of this model in predicting LAA thrombosis but also highlight its robustness in adapting to varying clinical datasets. Most importantly, this model enables non-invasive, convenient early risk assessment for AF patients using routine TTE and clinical data, offering significant clinical value.

The superior performance of the Logistic Regression model underscores its critical role in screening high-risk AF patients, particularly in those unable to undergo TEE. By incorporating clinical risk scores (such as CHA2DS2-VASc), echocardiographic features (such as LAD and LVEF), and biochemical markers (such as D-dimer), this model precisely identifies patients at higher risk of thrombosis, providing reliable guidance for early intervention. Clinically, this model not only aids in efficiently screening high-risk patients but also assists physicians in making individualized treatment decisions, ultimately reducing the incidence of thrombotic complications in AF patients. Furthermore, key features identified by the model, such as LAD, D-dimer, heart failure, and AF type, further elucidate the multifactorial mechanisms of LAA thrombosis and provide important biomarkers and clinical parameters for future risk assessments. Overall, the Logistic Regression model not only improves the accuracy of LAA thrombosis screening but also offers a low-cost, high-efficiency screening tool for primary care settings and resource-limited regions, with broad clinical applicability and the potential to enhance the management of AF patients and reduce thrombotic events.

### 4.1 Limitation

This study has some limitations. First, the limitations of potential feature selection bias, regional population bias from the single-center design, and the absence of advanced visualization methods (e.g., SHAP, LIME). Second, the model relies solely on TTE and clinical data and does not incorporate other imaging modalities or biomarkers, which may restrict the optimization potential of the model. Future studies should incorporate multicenter validation, dynamic follow-up data, and multimodal imaging to enhance the model’s robustness and clinical utility.

## 5 Conclusion

This study presents a novel, non-invasive LAA thrombosis prediction model using machine learning, integrating clinical data and TTE. The Logistic Regression model shows superior diagnostic performance and prospective stability, particularly for AF patients unable to undergo TEE. This model provides a powerful tool for early intervention and personalized treatment, reducing thrombotic complications. Future multi-center validation and model optimization will further enhance its clinical application value.

## Data Availability

The raw data supporting the conclusions of this article will be made available by the authors, without undue reservation.
