# Correction: A new integrated machine learning model: application to improve the accuracy of predicting left atrial appendage thrombus in patients with non-valvular atrial fibrillation

**DOI:** 10.3389/fmed.2025.1718394

**Published:** 2025-10-14

**Authors:** Frontiers Production Office

**Affiliations:** Frontiers Media SA, Lausanne, Switzerland

**Keywords:** non-valvular atrial fibrillation, transthoracic echocardiography, left atrial appendage thrombosis, machine learning models, transesophageal echocardiography

The conflict of interest statement was erroneously given as “YL was employed by Yizhun Medical AI Co. Ltd. The remaining authors declare that the research was conducted in the absence of any commercial or financial relationships that could be construed as a potential conflict of interest.”

The correct conflict of interest statement is “YL was employed by Yizhun Medical AI Co. Ltd. The remaining authors declare that the research was conducted in the absence of any commercial or financial relationships that could be construed as a potential conflict of interest. The handling editor DZ declared a shared parent affiliation of Xi'an Jiaotong University with the author HH at the time of review.”

The original version of this article has been updated.

